# Adipocyte-specific GPRC6A ablation promotes diet-induced obesity by inhibiting lipolysis

**DOI:** 10.1016/j.jbc.2021.100274

**Published:** 2021-01-09

**Authors:** Satoru Mukai, Akiko Mizokami, Takahito Otani, Tomomi Sano, Miho Matsuda, Sakura Chishaki, Jing Gao, Tomoyo Kawakubo-Yasukochi, Ronghao Tang, Takashi Kanematsu, Hiroshi Takeuchi, Eijiro Jimi, Masato Hirata

**Affiliations:** 1 OBT Research Center, Faculty of Dental Science, Kyushu University, Fukuoka, Japan; 2 Department of Health and Nutrition care, Faculty of Allied Health Sciences, University of East Asia, Shimonoseki, Japan; 3 Division of Functional Structure, Department of Morphological Biology, Fukuoka Dental College, Fukuoka, Japan; 4 Department of Cell Biology and Pharmacology, Faculty of Dental Science, Kyushu University, Fukuoka, Japan; 5 Laboratory of Molecular and Cellular Biochemistry, Faculty of Dental Science, Kyushu University, Fukuoka, Japan; 6 Division of Applied Pharmacology, Kyushu Dental University, Kitakyushu, Japan; 7 Oral Medicine Research Center, Fukuoka Dental College, Fukuoka, Japan

**Keywords:** adipocyte, adipose triglyceride lipase (ATGL), forkhead box O1 (FoxO1), G protein–coupled receptor, lipolysis, obesity, GPRC6A, ATGL, adipose triglyceride lipase, db-cAMP, dibutyryl-cAMP, eWAT, epididymal white adipose tissue, FoxO1, forkhead box O1, GluOC, uncarboxylated form of osteocalcin, GTT, glucose tolerance test, HFS, high-fat and high-sucrose, HSL, hormone-sensitive lipase, IRF4, interferon regulatory factor 4, NEFA, nonesterified fatty acid, PKA, protein kinase A, PKI, protein kinase A inhibitor, PTT, pyruvate tolerance test, RER, respiratory exchange ratio

## Abstract

The G protein–coupled receptor GPRC6A regulates various physiological processes in response to its interaction with multiple ligands, such as extracellular basic amino acids, divalent cations, testosterone, and the uncarboxylated form of osteocalcin (GluOC). Global ablation of GPRC6A increases the susceptibility of mice to diet-induced obesity and related metabolic disorders. However, given that GPRC6A is expressed in many tissues and responds to a variety of hormonal and nutritional signals, the cellular and molecular mechanisms underlying the development of metabolic disorders in conventional knockout mice have remained unclear. On the basis of our previous observation that long-term oral administration of GluOC markedly reduced adipocyte size and improved glucose tolerance in WT mice, we examined whether GPRC6A signaling in adipose tissue might be responsible for prevention of metabolic disorders. We thus generated adipocyte-specific GPRC6A knockout mice, and we found that these animals manifested increased adipose tissue weight, adipocyte hypertrophy, and adipose tissue inflammation when fed a high-fat and high-sucrose diet compared with control mice. These effects were associated with reduced lipolytic activity because of downregulation of lipolytic enzymes such as adipose triglyceride lipase and hormone-sensitive lipase in adipose tissue of the conditional knockout mice. Given that, among GPR6CA ligands tested, GluOC and ornithine increased the expression of adipose triglyceride lipase in cultured 3T3-L1 adipocytes in a manner dependent on GPRC6A, our results suggest that the constitutive activation of GPRC6A signaling in adipocytes by GluOC or ornithine plays a key role in adipose lipid handling and the prevention of obesity and related metabolic disorders.

Obesity is characterized by excessive triglyceride storage in adipocytes and nonadipose tissues, such as the liver, skeletal muscle, and heart (1). It is a risk factor for the development of chronic conditions, such as type 2 diabetes mellitus, cardiovascular disease, and certain types of cancer, and therefore has a substantial impact on both the quality of life of affected individuals and national health care costs (2). The major underlying cause of obesity is an imbalance between energy intake and expenditure. A shift in the equilibrium away from triglyceride breakdown and toward triglyceride synthesis thus leads to the development and progression of obesity.

The G protein–coupled receptor GPRC6A has been implicated as a master regulator of metabolic processes (3). It is expressed in many tissues (4), including the pancreas, liver, skeletal muscle, small intestine, brain, and white adipose tissue, and is activated by multiple ligands, including basic amino acids, testosterone, various cations, and the bone-derived uncarboxylated form of osteocalcin (GluOC) (5, 6, 7, 8). Global deletion of *Gprc6a* in mice induces metabolic disorders, such as hyperglycemia, glucose intolerance, insulin resistance, hepatic steatosis, osteopenia, and feminization of males (9). Another line of GPRC6A-deficient mice manifested an increased susceptibility to high-fat diet-induced obesity and related metabolic disorders (10). Moreover, mice lacking GPR6CA specifically in pancreatic β-cells were found to be glucose intolerant as a result of the impairment of β-cell proliferation and insulin production (11). Furthermore, a study on liver-specific GPRC6A knockout mice revealed that GPRC6A signaling in the liver regulates glucose and fat metabolism directly and indirectly by regulating fibroblast growth factor-21 production (12). These observations have suggested that GPRC6A is a key regulator of energy homeostasis, given that it is expressed in many tissues and responds to a variety of hormonal and nutritional signals; however, the cellular and molecular mechanisms underlying the metabolic disorders that are characteristic of conventional knockout mice remain unclear.

We have previously shown that long-term oral administration of the GPRC6A ligand GluOC resulted in a marked reduction in adipocyte size and improved glucose tolerance in WT female mice (13, 14). We also found that treatment of 3T3-L1 adipocytes with GluOC resulted in the GPRC6A-dependent activation of extracellular signal–regulated kinase, expression of peroxisome proliferator–activated receptor γ, and production of adiponectin (13), which play a key role in adipocyte differentiation and glucose metabolism. GluOC stimulation also induced expression of the transcription factor forkhead box O1 (FoxO1) and that of its target gene for adipose triglyceride lipase (ATGL), resulting in the upregulation of lipolysis (15). These observations thus suggested that adipose tissue might be the main site of action for GPRC6A in the prevention of metabolic disturbances. We have therefore now generated adipocyte-specific GPRC6A knockout (adG6AKO) mice in order to explore the tissue-specific mechanism by which GPRC6A regulates global energy homeostasis. We found that adipose tissue weight, adipocyte size, and the extent of adipose tissue inflammation were all more pronounced in adG6AKO mice that were fed a high-fat and high-sucrose (HFS) diet than in control mice. The increased adiposity of adG6AKO mice was attributable to reduced lipolytic activity associated with the downregulation of lipolytic enzymes, including ATGL and hormone-sensitive lipase (HSL). Our results thus suggest that constitutive GPRC6A signaling in adipocytes induced by physiologically relevant hormones and nutrition-related factors contributes to the prevention of obesity and related metabolic disorders.

## Results

### Adipocyte-specific ablation of GPRC6A sensitizes male mice to diet-induced obesity

**Figure 1:**
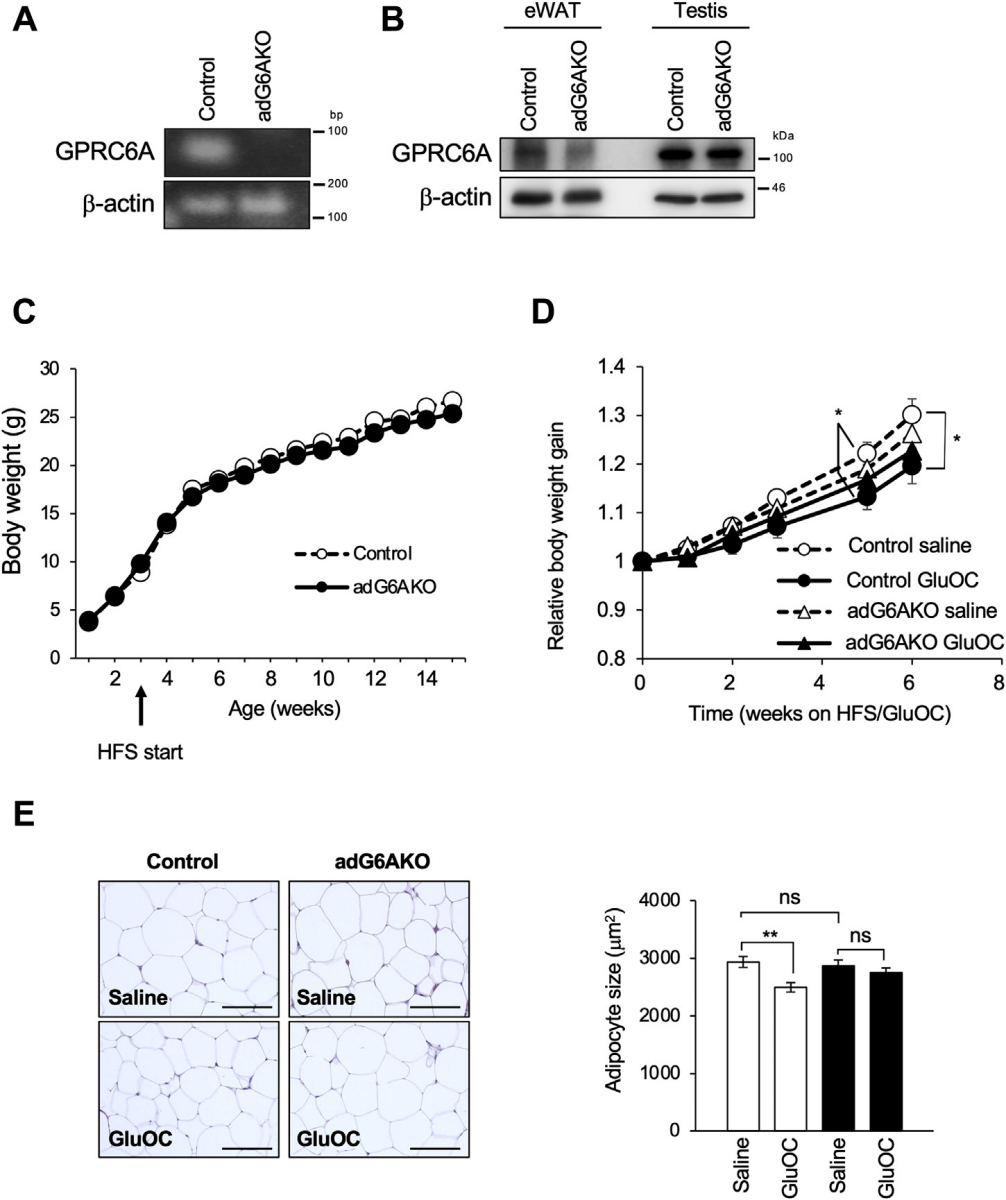
**GluOC administration in adipocyte-specific GPRC6A knockout female mice does not reduce adipocyte size.***A*, RT-PCR analysis of GPRC6A and β-actin (internal control) mRNAs in isolated adipocytes from eWAT of control (Gprc6a^fl/fl^) and adG6AKO mice. *B*, immunoblot analysis of GPRC6A and β-actin (loading control) in eWAT and testis (positive control) of control and adG6AKO mice. *C*, time course of body weight for control and adG6AKO mice during maintenance on the HFS diet for 12 weeks, beginning at 3 weeks of age. Error bars are omitted for clarity. *D*, time course of body weight for control and adG6AKO mice. GluOC (10 ng/g) or saline was administered daily for 6 weeks, beginning at 8 weeks of age. HFS feeding was started at the same time. *E*, H&E staining of eWAT from control and adG6AKO mice (*left*) and the determination of adipocyte area for 50 adipocytes per slide and at least three sections for each mouse (*right*). The bars represent 50 μm. All data are the mean ± SEM for 8 to 10 mice per group. ∗*p* < 0.05, ∗∗*p* < 0.01 for indicated comparison by two-way ANOVA (*D*) and Student's *t*-test (*E*). eWAT, epididymal white adipose tissue; GluOC, uncarboxylated osteocalcin, HFS, high-fat and high-sucrose.

adG6AKO mice were generated by crossing mice homozygous for a floxed allele of Gprc6a (Gprc6a^fl/fl^ mice) with Fabp4–Cre mice, which express Cre recombinase under the control of the adipose tissue–specific promoter of Fabp4 (Fig. S1). Ablation of GPRC6A, specifically in adipose tissue, was evaluated by immunoblot and RT-PCR analyses. GPRC6A was virtually undetectable at both the protein and mRNA levels in adipocytes isolated from epididymal white adipose tissue (eWAT) of adG6AKO mice (Fig. 1, *A* and *B*), whereas the amount of GPRC6A protein in the testis, in which GPRC6A is highly expressed (8), did not differ between adG6AKO and control (Gprc6a^fl/fl^) mice (Fig. 1*B*). This result was indicative of successful adipocyte-targeted Gprc6a depletion. Very little contamination of macrophages, capillaries, and others in eWAT appeared to provide some positive signals, particularly in protein blots.

Body weight did not differ significantly between control and adG6AKO mice of both sexes maintained on a normal diet (Fig. S2, *A* and *B*), which is consistent with previous results for whole-body GPRC6A knockout mice (16). Therefore, we examined the effects of feeding the mice with an HFS diet, beginning immediately after the lactation period (3 weeks of age).

We found that body weight gain was similar between the genotypes in female mice in the HFS diet setting (Fig. 1*C*). We then tested the effects of long-term GluOC treatment in these mice. Four weeks of GluOC treatment slightly reduced body weight gain only in the control mice (Fig. 1*D*). Furthermore, consistent with our previous studies (13, 14), the size of the adipocytes was significantly reduced in control mice, but not in adG6AKO mice, after 4 weeks of treatment with GluOC (Fig. 1*E*).

**Figure 2:**
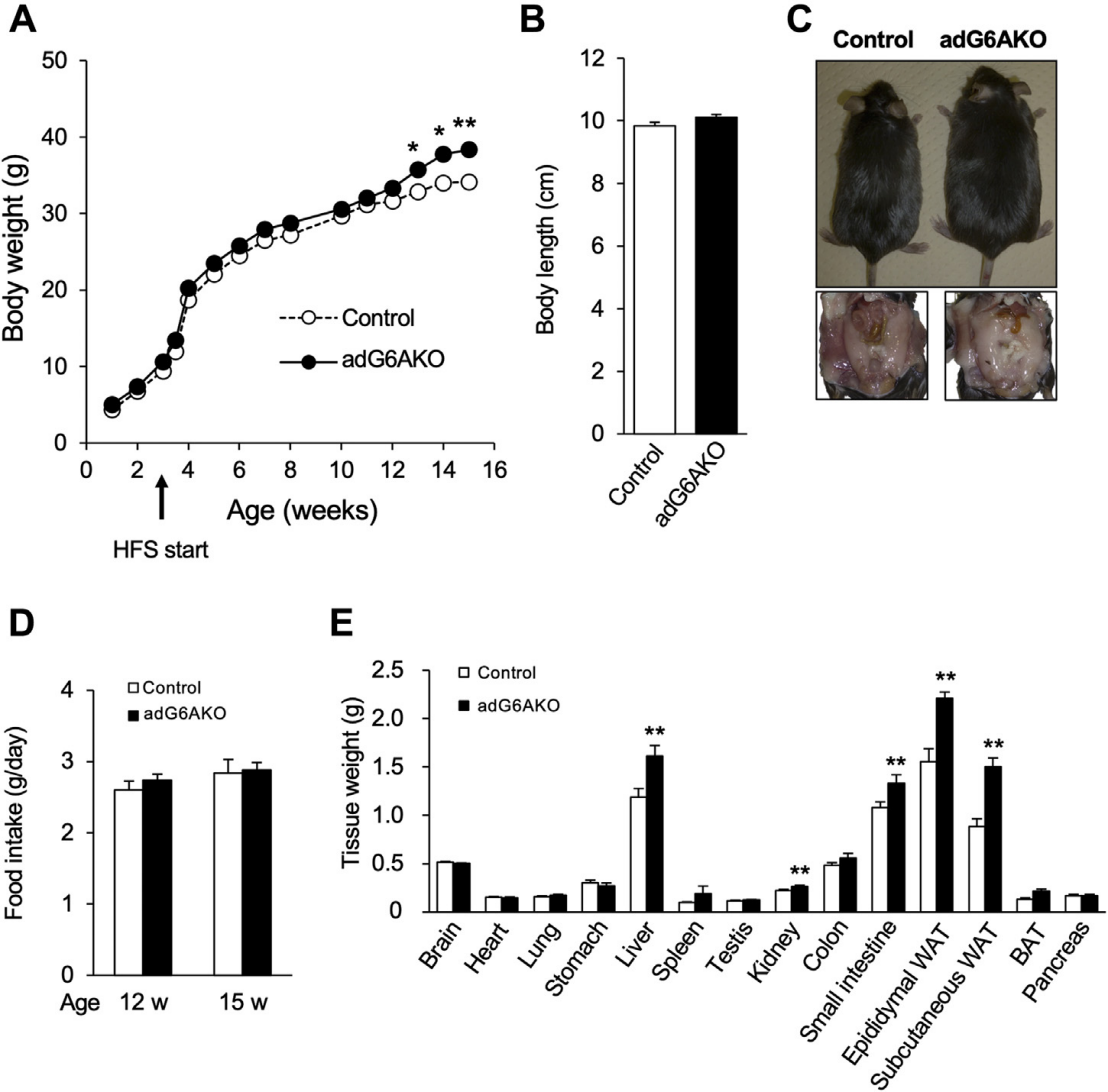
**Adipocyte-specific ablation of GPRC6A sensitizes male mice to diet-induced obesity.***A*, time course of body weight for control and adG6AKO mice during maintenance on the HFS diet for 12 weeks, beginning at 3 weeks of age. Error bars are omitted for clarity. *B*, body length of the control and adG6AKO mice at 15 weeks of age. *C*, representative appearance and epididymal adipose tissues of control and adG6AKO mice fed with HFS for 15 weeks. *D*, food intake measured over 2-week intervals, ending at 12 and 15 weeks of age. *E*, organ and tissue weights after maintenance of mice on the HFS diet for 15 weeks. All data are the mean ± SEM for 8 to 10 mice per group. ∗*p* < 0.05, ∗∗*p* < 0.01 *versus* the corresponding value for control mice by two-way ANOVA (*A*) and Student's *t* test (*E*). BAT, interscapular brown adipose tissue; HFS, high-fat and high-sucrose; WAT, white adipose tissue.

On the other hand, the same experiments using male mice revealed that after 15 weeks of HFS feeding, adG6AKO mice were larger with more fat accumulation in the epididymal adipose tissue than that in control mice (Fig. 2*A*). Body weight did not differ between the two genotypes until 13 weeks of age, when that of adG6AKO mice became significantly greater than that of the control mice; this result was observed despite the fact that the anal–nasal body length was similar in both groups of animals (Fig. 2, *B* and *C*). Food intake did not differ between the two genotypes either before (12 weeks of age) or after (15 weeks of age) adG6AKO mice started to gain more weight than control mice (Fig. 2*D*). After maintenance on the HFS diet for 12 weeks, the weights of epididymal and subcutaneous adipose tissue, as well as of the liver, kidney, and small intestine, were significantly greater in adG6AKO mice than in control mice (Fig. 2*E*).

### Histological analysis and metabolic assessment of adG6AKO mice

**Figure 3:**
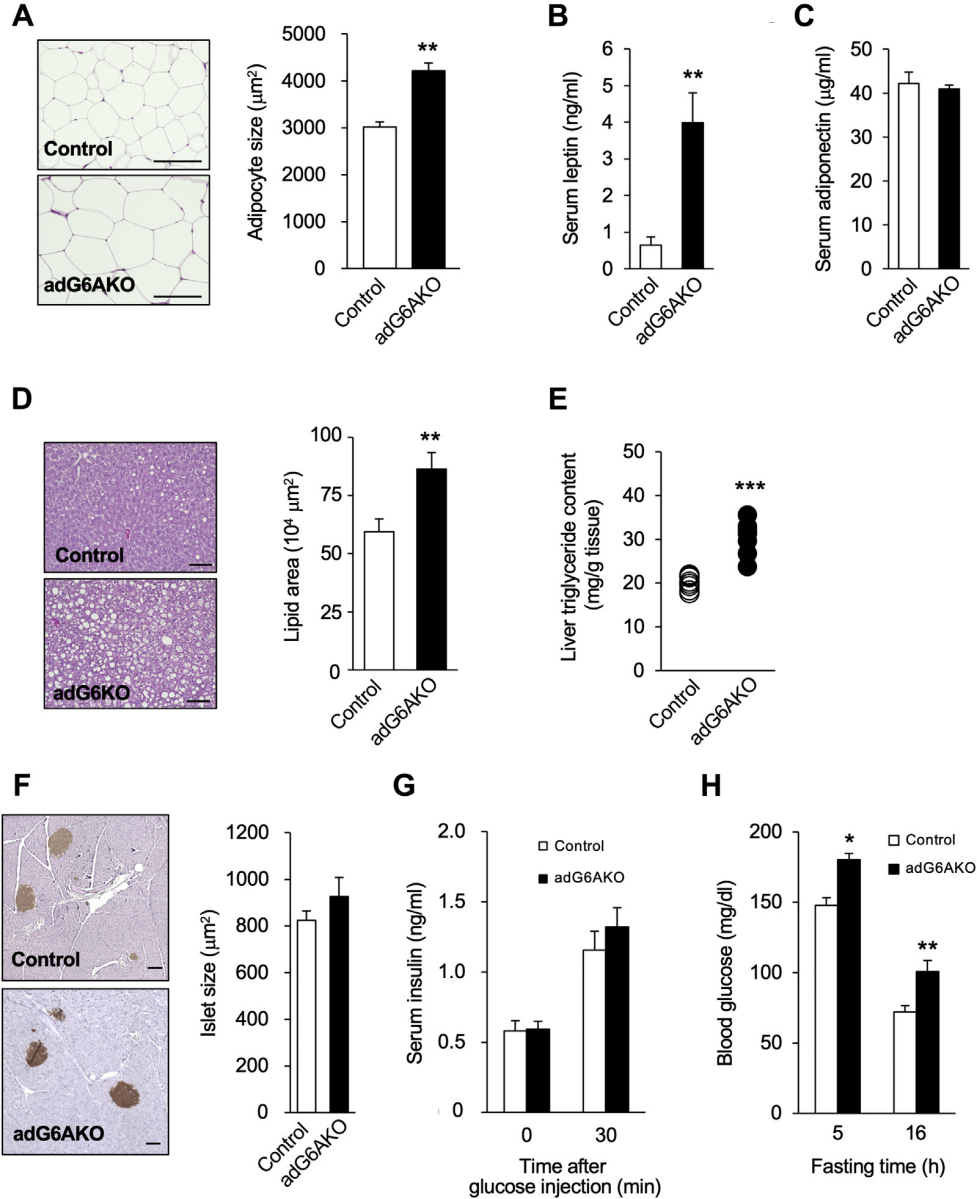
**Histological analysis of adG6AKO male mice maintained on the HFS diet for 15 weeks.***A*, H&E staining of eWAT from control and adG6AKO mice (*left*) and determination of adipocyte area for 50 adipocytes per slide and at least three sections for each mouse (*right*). The bars represent 50 μm. *B* and *C*, serum concentrations of leptin and adiponectin, respectively, measured in the fed state. *D*, H&E staining of liver sections from control and adG6AKO mice (*left*) and determination of lipid area in at least three sections for each mouse (*right*). The bars represent 100 μm. *E*, liver triglyceride content was measured after 15 weeks of HFS feeding. *F*, immunostaining for insulin in pancreatic sections counterstained with hematoxylin (*left*) and determination of islet area in at least three sections for each mouse (*right*). The bars represent 100 μm. *G*, serum insulin levels after deprivation of food for 16 h and at 30 min after subsequent intraperitoneal injection of glucose (1 g/kg). *H*, blood glucose levels after deprivation of food for 5 or 16 h. All quantitative data are the mean ± SEM for 8 to 10 mice per group. ∗*p* < 0.05, ∗∗*p* < 0.01, ∗∗∗*p* < 0.001 *versus* the corresponding value for control mice (Student's *t* test). eWAT, epididymal white adipose tissue; HFS, high-fat and high-sucrose.

Histological analysis revealed that adipocytes in eWAT of adG6AKO mice were significantly larger than those in control mice after maintenance on the HFS diet for 15 weeks (Fig. 3*A*). Consistent with this finding, the serum concentration of leptin was significantly increased (Fig. 3*B*), whereas that of adiponectin was unchanged (Fig. 3*C*) in adG6AKO mice. The size of adipocytes in adG6AKO mice fed a normal diet was also significantly greater than that in control mice at 25 weeks of age (Fig. S2*C*). Histological analysis of the liver showed that ectopic fat accumulation, which is often associated with obesity, and liver triglyceride content were greater in adG6AKO mice that were fed the HFS diet for 15 weeks than in control mice (Fig. 3, *D* and *E*). The morphology and size of pancreatic islets as well as the serum concentrations of insulin under both basal and glucose-stimulated conditions were similar in mice of the two genotypes that were fed the HFS diet for 15 weeks (Fig. 3, *F* and *G*). However, blood glucose levels after food deprivation for 5 or 16 h were significantly higher in the adG6AKO mice (Fig. 3*H*), suggesting global resistance to insulin in the mutant animals. A similar increase in the fasting blood glucose concentration was apparent in the adG6AKO mice that were fed a normal diet (Fig. S2*D*).

### Adipocyte-specific ablation of GPRC6A promotes glucose intolerance

**Figure 4:**
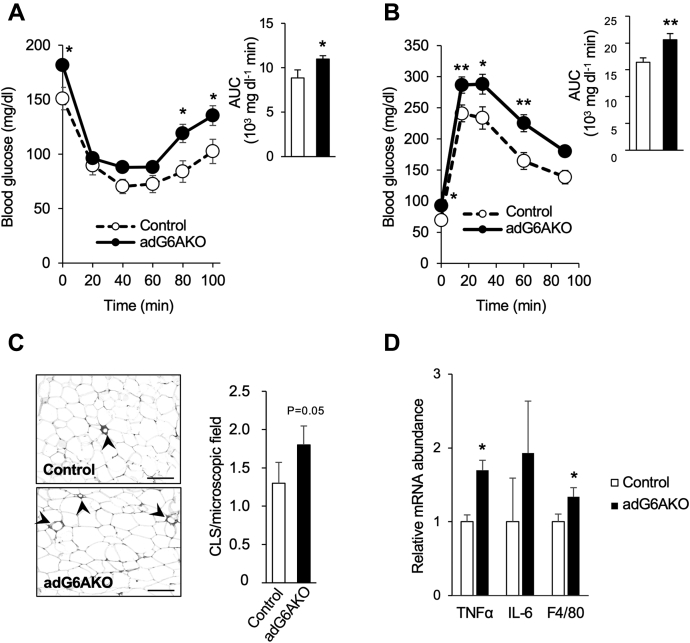
**Adipocyte-specific ablation of GPRC6A promotes glucose intolerance and adipose tissue inflammation.** Control and adG6AKO mice were subjected to an insulin tolerance test (*A*) or to an intraperitoneal glucose tolerance test (*B*) after maintenance on the HFS diet for 13 or 12 weeks, respectively. The area under the curve (AUC) is shown in each inset. *C*, immunohistochemical staining of the macrophage marker F4/80 in eWAT from control and adG6AKO mice maintained on the HFS diet for 15 weeks (*left*) as well as quantitation of crownlike structures (CLS) in at least three sections for each mouse (*right*). Arrowheads indicate F4/80-positive macrophage infiltration and formation of CLS. The bars represent 100 μm. *D*, quantitative RT-PCR analysis of relative mRNA abundance for the indicated inflammatory molecules in eWAT of control and adG6AKO mice as in *C*. Results were normalized to the amount of β-actin mRNA. All data are the mean ± SEM for 8 to 10 mice per group. ∗*p* < 0.05, ∗∗*p* < 0.01 *versus* the corresponding value for control mice (two-way ANOVA followed by Bonferroni's post hoc test [*A* and *B*] or Student's *t* test [*C* and *D*]). eWAT, epididymal white adipose tissue; HFS, high-fat and high-sucrose; IL-6, interleukin 6; TNF, tumor necrosis factor.

Increased adiposity and ectopic lipid accumulation in the liver can trigger insulin resistance and consequent impairment of systemic glucose handling (17). Indeed, an insulin tolerance test revealed that adG6AKO mice that were fed the HFS diet for 13 weeks were resistant to the glucose-lowering effect of insulin compared with their control counterparts (Fig. 4*A*). An intraperitoneal glucose tolerance test (GTT) also showed that the increase in blood glucose levels induced by glucose injection was significantly higher, and subsequent glucose clearance appeared slower in the mutant mice compared with the control mice (Fig. 4*B*).

Low-grade inflammation in adipose tissue is characterized by macrophage infiltration and the formation of crownlike structures and is a crucial risk factor for insulin resistance. This inflammation was increased in eWAT of adG6AKO mice, as assessed by staining for the macrophage marker F4/80 (18) (Fig. 4*C*). Consistent with this inflammatory phenotype, the abundance of mRNAs for the proinflammatory markers tumor necrosis factor α and F4/80 was increased in eWAT of mutant mice (Fig. 4*D*).

### Adipocyte-specific ablation of GPRC6A induces adipocyte hypertrophy because of impairment of lipolysis

**Figure 5:**
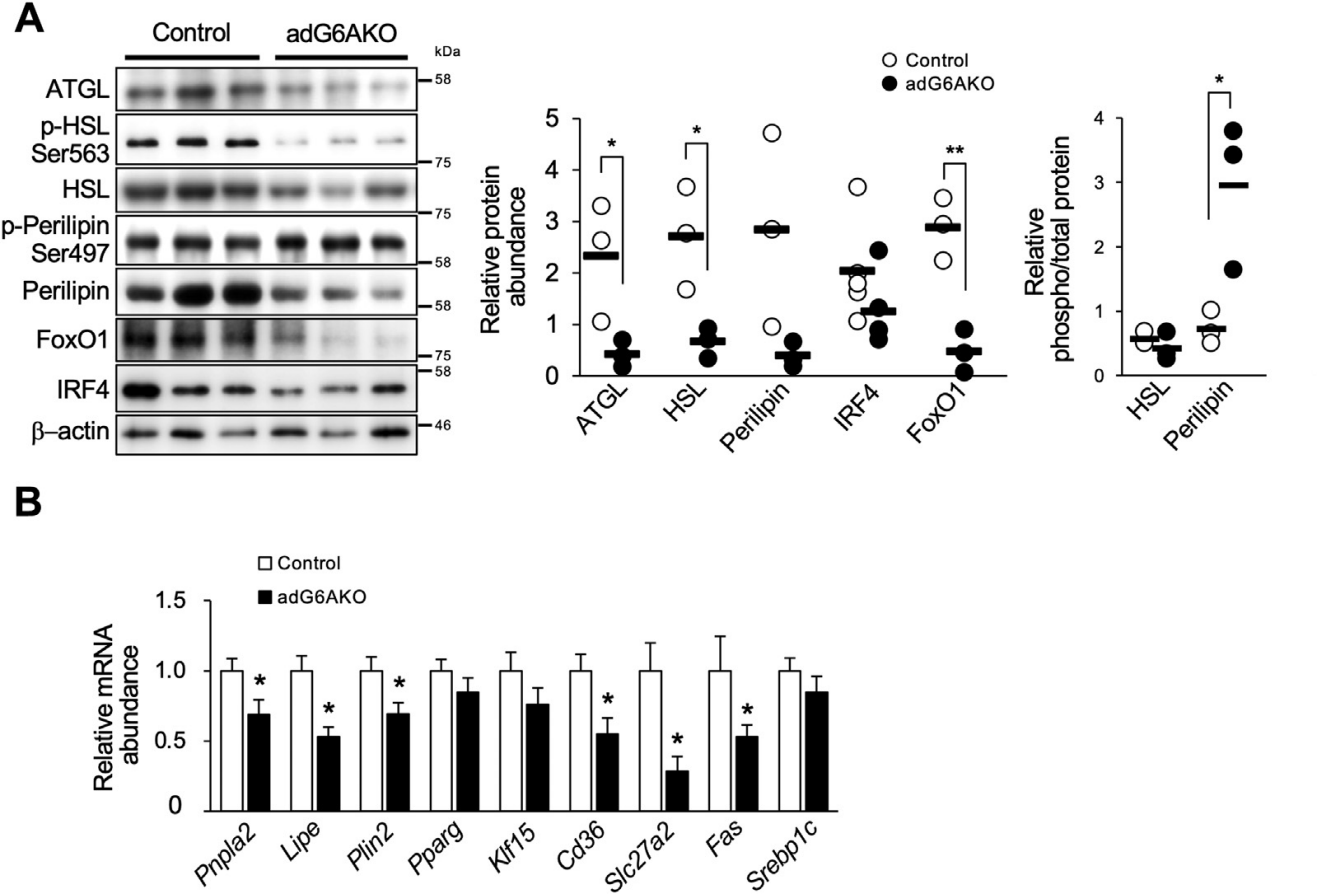
**Adipocyte-specific ablation of GPRC6A induces adipocyte hypertrophy because of impairment of lipolysis.***A*, immunoblot analysis of total or the indicated phosphorylated (p-) forms of adipose triglyceride lipase (ATGL), hormone-sensitive lipase (HSL), perilipin, forkhead box O1 (FoxO1), and interferon regulatory factor 4 (IRF4) in eWAT of control and adG6AKO mice that were fed the HFS diet for 15 weeks. Representative blots are shown for three mice of each genotype as well as quantitative data from multiple blots for the relative abundance of each protein normalized by that of β-actin and for the relative phosphorylated/total protein ratio for HSL and perilipin. *B*, quantitative RT-PCR analysis of relative mRNA abundance for the indicated genes in eWAT of control or adG6AKO mice that were fed the HFS diet, as in *A*. Results were normalized to the amount of β-actin mRNA. All quantitative data are the mean ± SEM for 8 to 10 mice per group. ∗*p* < 0.05, ∗∗*p* < 0.01 *versus* the corresponding value for control mice (Student's *t* test). eWAT, epididymal white adipose tissue.

Expansion of adipocytes can result from either increased lipogenesis or suppression of lipolysis. We recently showed that stimulation of 3T3-L1 adipocytes with the GPRC6A ligand GluOC increased the expression of both ATGL and its upstream transcription factor FoxO1 (15). To examine whether GPRC6A activation has similar effects in the adipocytes of mice, we isolated eWAT of adG6AKO and control mice that were fed the HFS diet. Immunoblot analysis revealed that the abundance of lipolysis-related proteins, including ATGL, HSL, perilipin, and FoxO1, was markedly downregulated in eWAT of adG6AKO mice compared with that of control mice (Fig. 5*A*). The extent of HSL phosphorylation at Ser^563^, which promotes lipolysis, was unchanged in adipocytes of the mutant mice; meanwhile, that of perilipin at Ser^497^, which triggers lipolysis by leading to the activation of ATGL, was increased (Fig. 5*A*). The abundance of interferon regulatory factor 4 (IRF4), a FoxO1-dependent transcription factor that regulates the expression of both ATGL and HSL (19), also tended to be reduced in adipocytes of adG6AKO mice, although this difference was not statistically significant (Fig. 5*A*). Immunoblot analysis also showed that the amounts of ATGL and HSL in eWAT were significantly reduced or tended to be reduced, respectively, in 25-week-old adG6AKO mice that were fed a normal diet compared with control mice (Fig. S2*E*). Consistent with this latter finding, the serum nonesterified fatty acid (NEFA) level after food deprivation for 4 h was significantly lower in the mutant mice (Fig. S2*F*).

We also examined the expression of lipolysis- and adipogenesis-related molecules at the mRNA level in the eWAT of adG6AKO and control mice that were fed the HFS diet (Fig. 5*B*). The expression of lipolysis-related genes, including *Pnpla2* (encoding ATGL), *Lipe* (encoding HSL), and *Plin2* (encoding perilipin), was significantly downregulated in eWAT of adG6AKO mice. On the other hand, expression of the adipogenic genes, *Pparg*, *Klf15*, and *Srebp1c*, did not differ between the two genotypes, suggesting that adipogenesis is not affected by Gprc6a deletion. Expression of *Cd36* and *Slc27a2*, both of which encode proteins that contribute to fatty acid uptake as well as that of the lipogenic gene *Fas* (encoding fatty acid synthase), was also significantly reduced in GPRC6A-deficient adipose tissue. These findings indicate that GPRC6A ablation in adipocytes results in the impairment of lipolysis and consequent adipocyte hypertrophy.

### Impairment of lipolysis because of adipocyte-specific ablation of GPRC6A leads to altered fuel utilization

**Figure 6:**
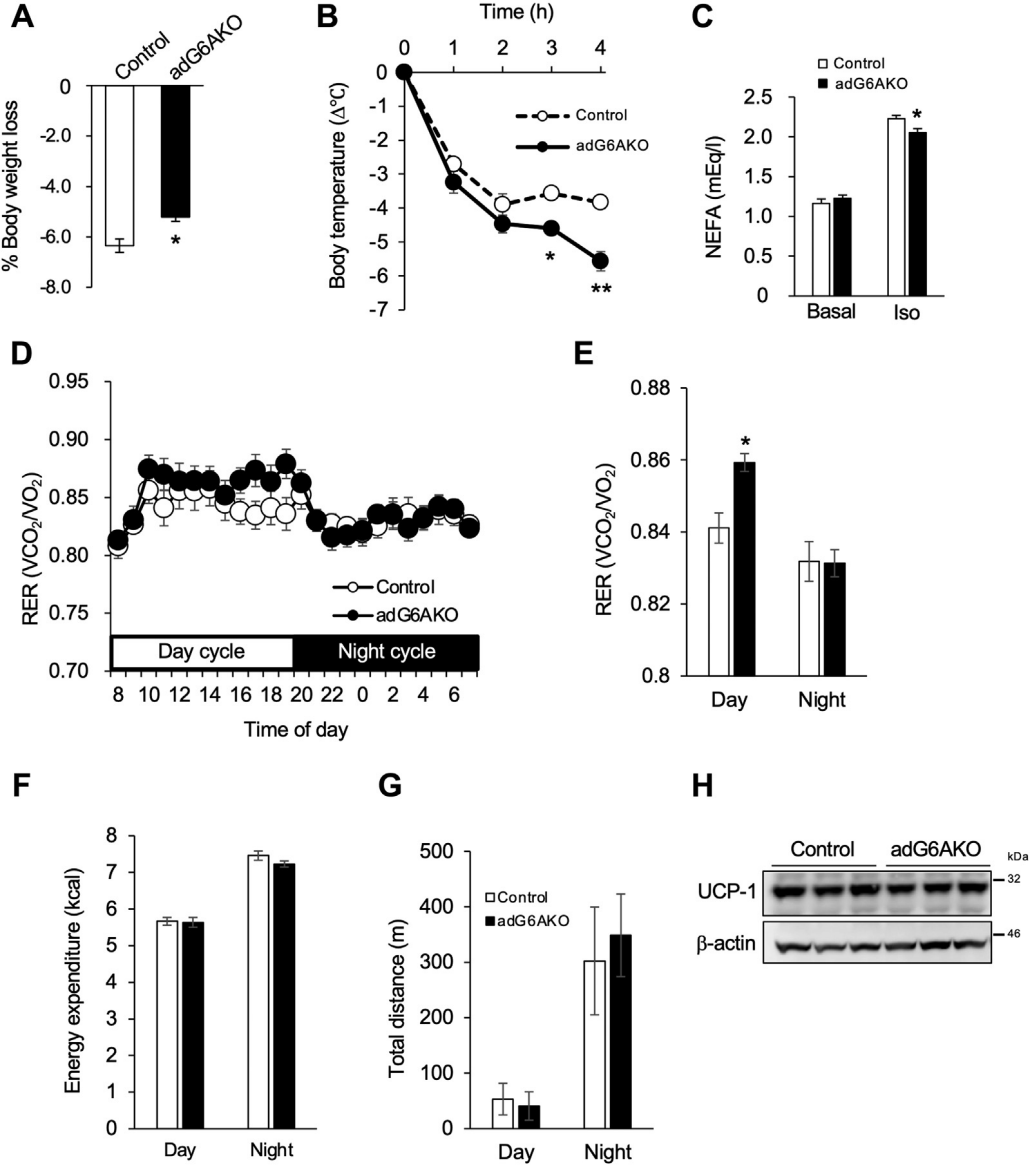
**Impairment of lipolysis because of adipocyte-specific ablation of GPRC6A leads to altered fuel utilization.***A*, response of control and adG6AKO mice to 24 h of fasting. *B*, decline in rectal temperature during cold exposure (4 °C). *C*, serum NEFA levels before and 15 min after intraperitoneal injection of isoproterenol (10 mg/kg) in food-deprived control and adG6AKO mice. D, respiratory exchange ratio (RER) calculated from oxygen consumption (VO_2_) and carbon dioxide production (VCO_2_) assessed by an indirect calorimetric system over a 24-h period with a 12-h light/dark cycle (day from 8 AM to 8 PM). *E*, average RER for day and night period. *F*, cumulative energy expenditure during daytime and nighttime. *H,* UCP1 expression in brown adipose tissue from control and adG6AKO mice. β-actin was used as a loading control. All quantitative data are the mean ± SEM from at least three independent experiments. ∗*p* < 0.05, ∗∗*p* < 0.01 for the indicated comparisons by one-way ANOVA (*B*) or Student's *t* test (*A*, *C*, and *E*). NEFA, nonesterified fatty acid; UCP-1, uncoupling protein 1.

To examine whether the suppression of lipolytic molecules in GPRC6A-deficient adipose tissue is actually reflected in the impairment of lipolysis in living mice, we examined control and adG6AKO mice in response to three situations that require lipolysis. First, we subjected the mice to prolonged fasting for 24 h and measured their weight loss. adG6AKO mice lost significantly less weight than control mice in this setting (Fig. 6*A*). Second, cold-induced thermogenesis was examined. Body temperatures at 25 °C were similar between control and adG6AKO mice (37.6 ± 0.09 and 37.7 ± 0.13, respectively; n = 10). However, at 4 °C, adG6AKO mice experienced a more severe decline in body temperature compared with their counterparts (Fig. 6*B*). Finally, we evaluated the catecholamine-stimulated release of NEFAs into the circulation. The isoproterenol-induced increase in the serum NEFA concentration was significantly attenuated in adG6AKO mice compared with that in control mice (Fig. 6*C*). The expression levels of β3-adrenergic receptor in the membrane fractions of WAT from control and mutant mice were similar, indicating no difference in β3-adrenergic receptor signaling (Fig. S3). These results suggest that the mutant mice showed lower metabolism, probably by using lipids.

The respiratory exchange ratio (RER) calculated from oxygen consumption and carbon dioxide production during the resting day cycle was significantly increased in adG6AKO mice, suggesting impaired fat utilization because of the reduction of lipolysis (Figs. 6, *D* and *E* and S4, *A* and *B*). Little difference of RER between daytime and nighttime in control mice might be due to high fat feeding. The change in energy partitioning was also supported by an increase in hepatic gluconeogenesis, as assessed by the pyruvate tolerance test (PTT; Fig. S4*C*). Cumulative energy expenditure and locomotor activity, assessed by measuring the total distances traveled over 24 h, were not altered between the two genotypes (Fig. 6, *F* and *G*). Uncoupling protein-1 expression levels in brown adipose tissue were not altered by GPRC6A ablation (Fig. 6*H*).

### Activation of GPRC6A signaling upregulates ATGL expression in 3T3-L1 adipocytes

**Figure 7:**
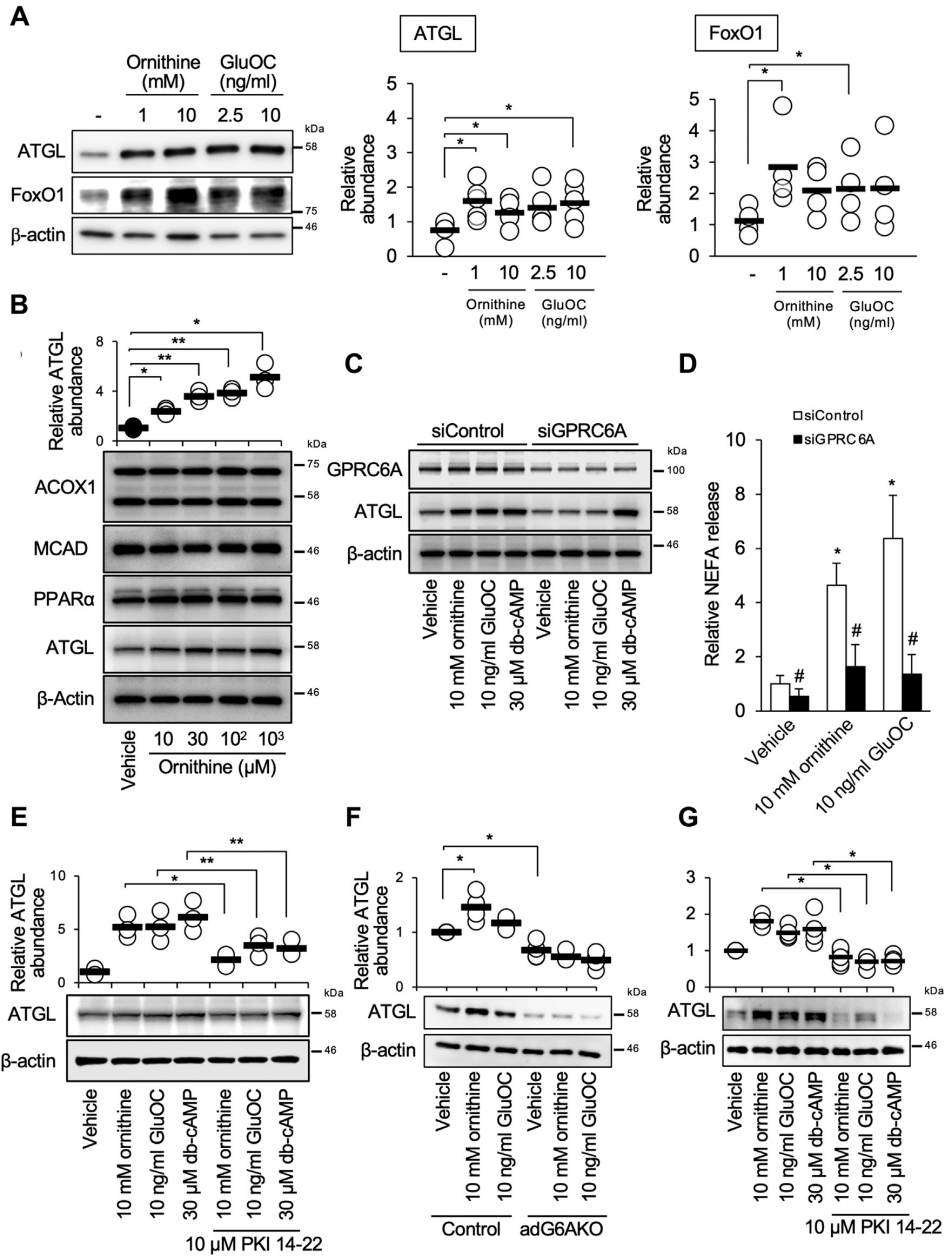
**Activation of GPRC6A signaling upregulates ATGL expression in 3T3-L1 adipocytes.***A*, immunoblot analysis of adipose triglyceride lipase (ATGL) and forkhead box O1 (FoxO1) in 3T3-L1 adipocytes stimulated with the indicated concentrations of ornithine or uncarboxylated osteocalcin (GluOC) for 8 h. *B* and *D*, cells were incubated with the indicated concentrations of ornithine for 8 h, after which the amount of free fatty acids released into the culture medium was measured (*D*). Cell lysates were subjected to immunoblot analysis of acyl-CoA oxidase-1 (ACOX1), medium-chain acyl-CoA dehydrogenase (MCAD), and peroxisome proliferator–activated receptor α (PPARα) (*B*). *C*, immunoblot analysis of GPRC6A and ATGL in 3T3-L1 adipocytes transfected with control or GPRC6A siRNAs and stimulated with the indicated concentrations of ornithine, GluOC, or dibutyryl-cAMP (db-cAMP) for 8 h. *E*, immunoblot analysis of ATGL in 3T3-L1 adipocytes stimulated with the indicated concentrations of ornithine, GluOC, or db-cAMP in the absence or the presence of protein kinase A (PKA) inhibitor (PKI) 14-22 for 8 h. *F*, immunoblot analysis of ATGL in explants of the adipose tissue from control and adG6AKO stimulated with the indicated concentrations of ornithine or GluOC for 24 h. *G*, immunoblot analysis of ATGL in explants of the adipose tissue from control mice stimulated with the indicated concentrations of ornithine, GluOC, or db-cAMP in the absence or the presence of PKI 14-22 for 24 h. Representative blots and quantitative data for relative protein abundance normalized to that of β-actin from multiple blots are shown. All quantitative data are the mean ± SEM from at least three independent experiments. ∗*p* < 0.05, ∗∗*p* < 0.01 for the indicated comparisons or #*p* < 0.05 *versus* the corresponding value for its corresponding control (one-way ANOVA).

To examine the mechanism by which GPRC6A signaling regulates lipolysis, we determined the effects of various GPRC6A ligands on the expression of lipolytic molecules in 3T3-L1 adipocytes. GPRC6A has been shown to be activated by multiple ligands, including arginine, ornithine, testosterone, and divalent cations, in addition to GluOC (20). Among the ligands tested (ornithine at 1 and 10 mM, GluOC at 2.5 and 10 ng/ml, arginine at 1 mM, testosterone at 10 nM, and Ca^2+^ at 1 mM), only ornithine and GluOC increased the abundance of ATGL (Figs. 7*A* and S5). Ornithine was also effective at lower concentrations (Fig. 7*B*). The effects of ornithine and GluOC on the expression of ATGL were abolished by siRNA-mediated depletion of GPRC6A (Fig. 7*C*). Accordingly, NEFA release into the culture medium was reduced by GPRC6A knockdown (Fig. 7*D*). The expression of enzymes responsible for both fatty acid β-oxidation and PPRAα, which regulates the transcription of these enzymes, was not affected by ornithine (Fig. 7*B*) or GluOC (15). Consistent with our previous observation that GluOC induced cAMP accumulation in a GPRC6A-dependent manner in 3T3-L1 adipocytes (13), we found that dibutyryl-cAMP (db-cAMP), a cell-permeable analog of cAMP, mimicked the effects of GluOC and ornithine on ATGL expression and restored ATGL expression in GPRC6A knockdown cells (Fig. 7*C*). The protein kinase A (PKA) inhibitor (PKI) 14-22 attenuated these effects (Fig. 7*E*).

Finally, we confirmed the effect of deficiency in GPRC6A signaling observed in 3T3-L1 adipocytes using *ex vivo* culture of adipose tissue from control and adG6AKO mice. Ornithine and GluOC increased the abundance of ATGL in control adipose tissue but had no effect on GPRC6A-deficient adipose tissue (Fig. 7*F*). We also confirmed that db-cAMP stimulated ATGL expression as ornithine, GluOC, and the PKA inhibitor PKI 14-22 attenuated these effects in control adipose tissue (Fig. 7*G*). These results indicate that GPRC6A signaling triggered by ornithine or GluOC might induce lipolysis by increasing the expression of ATGL in adipose tissue.

## Discussion

Here, we examined the role of GPRC6A expression in adipose tissue in the development of obesity. We found that adipocyte-specific Gprc6a deletion increased the susceptibility of mice to diet-induced obesity, with this effect being attributable to impaired lipolysis in adipose tissue and the consequent development of adipocyte hypertrophy. The obesity phenotype of adG6AKO mice was associated with insulin resistance, glucose intolerance, inflammation in adipose tissue, and hepatic steatosis. We therefore propose that GPRC6A in adipose tissue is required for the regulation of global energy homeostasis, especially in an obesogenic setting.

Two lines of global GPRC6A knockout mice have been shown to manifest an obese phenotype (9, 10). One line was also found to be characterized by the development of metabolic syndrome, feminization of males, and osteopenia, although the underlying mechanisms were not addressed (9). The obese phenotype of the second line was shown to be due to hyperphagia and reduced locomotion and was associated with increased circulating insulin and leptin levels, impairment of glucose metabolism, and an increase in the expression of the *Pomc* gene (encoding pro-opiomelanocortin) in the hypothalamus, with this latter effect likely being because of the increased circulating insulin and leptin levels (10). Given that Pomc expression in the hypothalamus reduces food intake and increases energy expenditure (21, 22), it was proposed that global GPRC6A knockout mice are resistant to the effects of Pomc-derived neuropeptides (10). We have now found that ablation of GPRC6A in adipose tissue had no effect on food intake despite an increase in the serum leptin concentration, with leptin having been shown to reduce appetite. Leptin resistance, in addition to impaired lipolysis, might contribute to the development of diet-induced obesity in adG6AKO mice.

The effect of adipose GPRC6A ablation on lipolysis was not as evident in female mice. This was probably because of the effect of estradiol, which makes females less susceptible to obesity (23). Long-term GluOC administration attenuated adipocyte expansion triggered by HFS diet feeding only in control female mice, consistent with our previous findings (13, 14). On the other hand, given that long-term GluOC administration induces insulin resistance in male mice because of an increase in circulating testosterone levels (14), it is likely that additional treatment with GluOC would be a more deteriorative additive to GPRC6A ablation.

ATGL and HSL are key lipolytic enzymes that act sequentially in the process of triglyceride breakdown in adipocytes (24, 25). We found that adG6AKO mice showed reduced expression levels of ATGL and HSL in adipose tissue, likely accounting at least in part for the impairment of lipolysis and consequent adipocyte expansion in these animals. Indeed, insufficient lipolysis has been associated with obesity in mice as well as in humans (19, 26). We recently showed that GluOC, an endogenous ligand for GPRC6A, increased FoxO1 expression in 3T3-L1 adipocytes (15). Here, we found that out of the known ligands for GPRC6A other than GluOC, only ornithine increased FoxO1 expression in 3T3-L1 adipocytes. The downregulation of FoxO1 expression in the adipose tissue of adG6AKO mice was thus possibly because of the absence of endogenous GluOC or ornithine signaling. This downregulation was likely responsible for the associated downregulation of ATGL expression, given that the *ATGL* gene is a direct target of FoxO1 (27). In addition to its direct transcriptional regulation of the *ATGL* gene, FoxO1 indirectly regulates the expression of ATGL and HSL in a manner dependent on IRF4 (19). IRF4 is a FoxO1-dependent transcription factor whose expression is highly restricted to immune cells and mature adipocytes (28). Together, these findings suggest that constitutive activation of GPRC6A signaling in adipose tissue by endogenous ligands such as GluOC and ornithine is required for FoxO1 and IRF4 expression and the consequent upregulation of the lipolytic enzymes ATGL and HSL. We also found that perilipin was hyperphosphorylated in the adipose tissue of adG6AKO mice, even though perilipin abundance was greatly downregulated. It is possible that this phosphorylation of perilipin is triggered by a signaling system that is activated as a compensatory mechanism.

Metabolic characterization of adG6AKO mice that were fed a normal diet revealed no apparent impairment of glucose handling (data not shown), although they did show a significant expansion of adipocytes and a slight increase in fasting blood glucose levels. However, when fed an HFS diet, adG6AKO mice manifested an increased fasting blood glucose level, glucose intolerance, and insulin resistance without any change in fasting or glucose-induced serum insulin levels compared with control mice. Hyperglycemia after fasting can be explained by altered fuel utilization because of impairment of lipolysis. Food intake is low, and thus the main source of energy is shifted to fatty acids during the daytime in mice. However, adG6AKO showed lower lipid metabolism because of reduced utilization of triglycerides as an energy source; therefore, the mutant mice develop greater hepatic gluconeogenesis in a compensatory manner, as assessed by the PTT.

In addition to increased adipose tissue weight, adG6AKO mice that were fed the HFS diet showed significant increases in the weight of the liver, kidney, and small intestine compared with control mice, suggesting ectopic lipid accumulation in the mutant animals. These effects of the HFS diet in adG6AKO mice were accompanied by significant downregulation of the expression of several genes related to fatty acid uptake or lipogenesis in adipose tissue, possibly as a result of negative feedback in response to excessive triglyceride storage in adipocytes. Impaired uptake of fatty acids in adipocytes might result in increased uptake in the liver and other organs and lead to ectopic lipid accumulation, as observed in adG6AKO mice that were fed the HFS diet. Given that ectopic lipid accumulation and chronic inflammation are associated with insulin resistance (1, 29), these observations suggest that the insulin resistance and glucose intolerance of adG6AKO mice that were fed the HFS diet are likely secondary to the excessive adiposity caused by impaired lipolysis and ectopic lipid accumulation.

GPRC6A has been implicated as a receptor for a wide range of ligands (3); however, among the ligands tested in this study, only ornithine and GluOC induced ATGL expression in 3T3-L1 adipocytes. This effect of both ornithine and GluOC was apparent at physiologically relevant concentrations (30, 31, 32, 33, 34, 35, 36), with the physiological concentration of GluOC or ornithine being ∼7 ng/ml (equivalent to 1.4 nM) in mice or ∼100 μM, respectively, in the serum of adult mice. Impairment of lipolysis in adG6AKO mice might therefore be a consequence of the inability of circulating GluOC and ornithine to activate GPRC6A signaling in adipose tissue. Basic amino acids activate GPRC6A at concentrations in the micromolar range (5, 20, 35). This effect is potentially also physiologically relevant, although arginine did not induce ATGL expression in 3T3-L1 adipocytes at a concentration of 1 mM in our experiments.

In summary, adipocyte-specific ablation of GPRC6A promoted the development of diet-induced obesity in mice by inhibiting lipolytic activity. Our data indicate that loss of GPRC6A in adipocytes is largely, if not completely, responsible for the diet-induced obesity apparent in mice with systemic GPRC6A deficiency, suggesting that constitutive activation of GPRC6A signaling in adipocytes plays a key role in adipose lipid handling.

## Experimental procedures

### Generation of adG6AKO mice

The mutant Gprc6a allele was generated in collaboration with Unitech (Chiba, Japan). A genomic fragment containing Gprc6a was obtained from a bacterial artificial chromosome clone (clone ID, RP23-299O9 or RP23-325K4; Roswell Park Cancer Institute). The targeting vector contained a selection cassette comprising a neomycin resistance gene (Neo) flanked by flippase recognition target sites, which were inserted downstream of Gprc6a exon 3, as well as loxP sites that were inserted upstream of exon 2 and downstream of the selection cassette (Fig. 1*A*). C57BL/6 mouse embryonic stem cells were transfected with the targeting vector by electroporation and then subjected to selection with G418. Homologous recombination within the Gprc6a locus was confirmed by Southern blot analysis of HindIII- or NheI-digested DNA with 5′ and 3′ external probes, respectively. Cells heterozygous for the targeted mutation were microinjected into blastocysts of C57BL/6 mice, and the resulting chimeric male offspring were bred with C57BL/6 female mice expressing the Flippase 1 gene in order to remove the Neo cassette. The resulting offspring were screened by PCR analysis of genomic DNA, and those harboring a Gprc6a allele with loxP-flanked exons 2 and 3 (Gprc6a^fl^) were mated with mice of the strain B6.Cg-Tg (Fabp4–cre)1Rev/J (The Jackson Laboratory), which express Cre recombinase under the control of the fatty acid–binding protein 4 (Fabp4) promoter. Fabp4–Cre;Gprc6a^fl/+^ mice were then interbred to generate Fabp4–Cre;Gprc6a^fl/fl^ (adG6AKO) mice. Mouse genotypes were determined by PCR analysis with the primers (forward and reverse, respectively) 5′-CATGACTCAGAGGAAAACATACAGG-3′ and 5′-AGGTAGTTATTC GGATCATCAGCTA-3′ for Fabp4-Cre and 5′-CAGGCTATACATGT TGCAGATTAGA-3′ and 5′-ATACAAAACAAGCATCCCTAATTGA-3′ for Gprc6a.

### Animal experiments

All mouse experiments were approved by the Animal Ethics Committee of Kyushu University (approval no. A19-174). Control (Gprc6a^fl/fl^) and adG6AKO mice were maintained at three to five per cage in a specific pathogen-free facility under a 12-h light/dark cycle. Age-matched male mice had unlimited access to normal chow (CRF-1; Oriental Yeast) or an HFS diet (F2HFHSD; Oriental Yeast) after the lactation period. The nutritional composition of the HFS diet has been described previously (37). The body weight of the mice was monitored weekly. For the measurement of food intake, singly housed mice were fed with a Roden CAFE Type M system (Oriental Yeast). For serum analysis, blood (maximum of 100 μl) was collected from the orbital plexus of animals anesthetized with sevoflurane. Serum prepared from the blood samples was assayed for leptin, adiponectin, and insulin using ELISA kits obtained from Fujifilm Wako, Merck-Millipore, and Mercodia, respectively.

### GluOC administration

Recombinant mouse GluOC was prepared and orally administered to 8-week-old female mice, as described previously (34, 38). HFS feeding was initiated at the same time. After 6 weeks of treatment, WAT from these mice was subjected to histomorphometric analysis.

### Isolation of adipocytes

eWAT of control and adG6AKO mice was minced and treated with collagenase (1 mg/ml) for 1 h at 37 °C, followed by centrifugation at 1000*g* for 5 min. The resulting top fraction containing mature adipocytes was collected, suspended in PBS, and centrifuged again. Mature adipocytes were then collected for immunoblotting and RT-PCR analyses.

### Histochemical analysis

Excised eWAT, liver, and pancreas specimens were fixed in 4% paraformaldehyde, dehydrated with a series of ethanol solutions, embedded in paraffin, and sectioned at a thickness of 6 μm. The sections were then depleted of paraffin, rehydrated with PBS, and stained with Mayer's H&E Y (Muto Pure Chemicals). For insulin and F4/80 staining, pancreas and adipose tissue sections were incubated overnight at 4 °C with rabbit monoclonal antibodies to insulin (1:1000 dilution of #4590; Cell Signaling Technology) and F4/80 (1:100 dilution; ab111101; Abcam), respectively, according to routine procedures, including quenching of endogenous peroxidase and antigen retrieval. Immune complexes were detected with biotinylated goat antibodies to rabbit IgG (1:1000 dilution; Vector Laboratories) and an ABC Elite Kit plus ImmPact DAB (Vector Laboratories), and the sections were counterstained with Mayer's hematoxylin (Muto Pure Chemicals). Images were acquired with a BIOREVO BZ-9000 microscope (Keyence), and adipocyte area or the area of white dots (indicative of lipid droplets) in liver sections were evaluated using BZ-Analyzer-II software (Keyence).

### Measurement of liver triglyceride content

The frozen liver was homogenized with water at a ratio of 1:2. Lipids were extracted by adding three times the amount of chloroform–methanol (1:2; v/v). After centrifugation, the extracts in the lower layer were collected, evaporated, and dissolved in isopropyl alcohol–Triton X-1000 (9:1; v/v). Triglyceride levels were measured using LabAssay TG kits (Fujifilm Wako) according to the manufacturer's instructions.

### RNA isolation and quantitative RT-PCR analysis

Total RNA was isolated from eWAT with the use of a ReliaPrep RNA Tissue Miniprep kit (Promega) and was subjected to RT for 2 h at 37 °C using a High-Capacity complementary DNA Reverse Transcription kit (Life Technologies). The resulting complementary DNA was subjected to real-time PCR analysis with KOD SYBR qPCR Mix (Toyobo) and a Takara PCR Thermal Cycler Dice Gradient instrument (Takara Bio). The amount of β-actin was used as an internal reference for each sample. The sequences of PCR primers are listed in Table S1.

### Metabolic assessment

A GTT and PTT was performed in mice that had been deprived of food for 16 h. Glucose (1 g/kg) or sodium pyruvate (1.5 g/kg) was administered intraperitoneally, and the blood glucose concentration was measured at various times thereafter using Free Style Lite Blood Glucose test strips (Abbott Laboratories). An insulin tolerance test was performed in mice that had been deprived of food for 5 h. Insulin (0.1 U/kg; Humulin R, Eli Lilly) was injected intraperitoneally, and the blood glucose concentration at various times thereafter was measured, as in the GTT.

### Cold exposure

Twenty-week-old control and adG6AKO mice fed with HFS for 17 weeks were individually housed at 4 °C without food and with free access to water. Rectal temperature was measured with a digital thermometer with a rectal probe (BAT-12; Phystemp) at the indicated times.

### Measurement of NEFAs

Mice were deprived of food for 5 h and then injected intraperitoneally with isoproterenol (10 mg/kg). Blood was collected from the orbital plexus before and 15 min after the injection. Serum isolated from the blood samples was assayed for NEFAs with the use of a LabAssay NEFA kit (Fujifilm Wako).

### Analysis of energy metabolism

Twenty-week-old control and adG6AKO mice fed with HFS for 17 weeks were subjected to metabolic analyses as described previously (39). Oxygen consumption and carbon dioxide production were measured using a computer-controlled open-circuit calorimetry system (Oxymax System, Columbus Instruments). The energy expenditure and RER were calculated. After metabolic analyses, the mice were subjected to locomotor activity analysis. Mice were housed in a cage (a square arena, 30 × 30 cm, with 40-cm high opaque walls). After a couple of days of habituation, locomotor activity was recorded using a video camera (HDR-XR550V; Sony) and analyzed with an ANY-maze video tracking system (Stoelting Co).

### Cell culture and adipocyte differentiation

3T3-L1 preadipocytes (JCRB9014) were obtained from the Japanese Collection of Research Bioresources (JCRB) Cell Bank (Osaka, Japan) and were maintained under a humidified atmosphere of 5% CO_2_ at 37 °C in Dulbecco's modified Eagle's medium (Sigma–Aldrich) supplemented with 10% calf serum, penicillin (100 U/ml), and streptomycin (0.1 mg/ml). Differentiation of preadipocytes into adipocytes was induced as described previously (13). Recombinant mouse GluOC was purified as described previously (34). Ornithine was obtained from Fujifilm Wako, and testosterone, myristoylated PKI 14-22 (PKA inhibitor), and db-cAMP were obtained from Sigma–Aldrich.

### RNA interference

For depletion of GPRC6A, 3T3-L1 adipocytes were transfected with siControl (Silencer Select Negative Control #1) or siGPRC6A (Silencer Select s102234) siRNAs (Thermo Fisher Scientific) with the use of Viromer Blue (Lipocalyx).

### Explant culture of mouse epididymal WAT

eWAT was removed from control or adG6AKO mice at 8 to 10 weeks of age into ice-cold PBS, rinsed, cut into 2-mm pieces, and cultured in Dulbecco's modified Eagle's medium supplemented with 10% fetal bovine serum in the presence or the absence of the indicated reagents.

### Immunoblot analysis

Immunoblot analysis was performed as described previously (13). In brief, equal amounts of protein were fractionated by SDS-PAGE, the separated proteins were transferred to a polyvinylidene difluoride membrane (Merck-Millipore), and the membranes were incubated with the primary antibodies listed in Table S2. Immune complexes were detected with horseradish peroxidase–conjugated secondary antibodies (Cell Signaling Technology) and an enhanced chemiluminescence system (Immunostar LD; Wako). Band intensities were quantified with the use of ImageJ software (National Institutes of Health).

### Statistical analysis

Quantitative data are presented as the mean ± SEM and were analyzed as indicated using Prism software, version 6.0 (GraphPad Software) and SPSS software, version 27.0 (IBM SPSS Inc). A *p*-value of <0.05 was considered statistically significant.

## Data availability

All data are presented in the article or are available upon request. Please contact Akiko Mizokami (akiko-k@dent.kyushu-u.ac.jp).

## Conflict of interest

The authors declare that they have no conflicts of interest with the contents of this article.
