# Supplementary material for: Adipocyte-specific GPRC6A ablation promotes diet-induced obesity by inhibiting lipolysis
Source: J Biol Chem. 2021 Jan 9;296:100274. doi: 10.1016/j.jbc.2021.100274 (PMC7949034; doi:10.1016/j.jbc.2021.100274)
Supplement: Figures and Tables [file mmc1.pdf]

Supporting Information

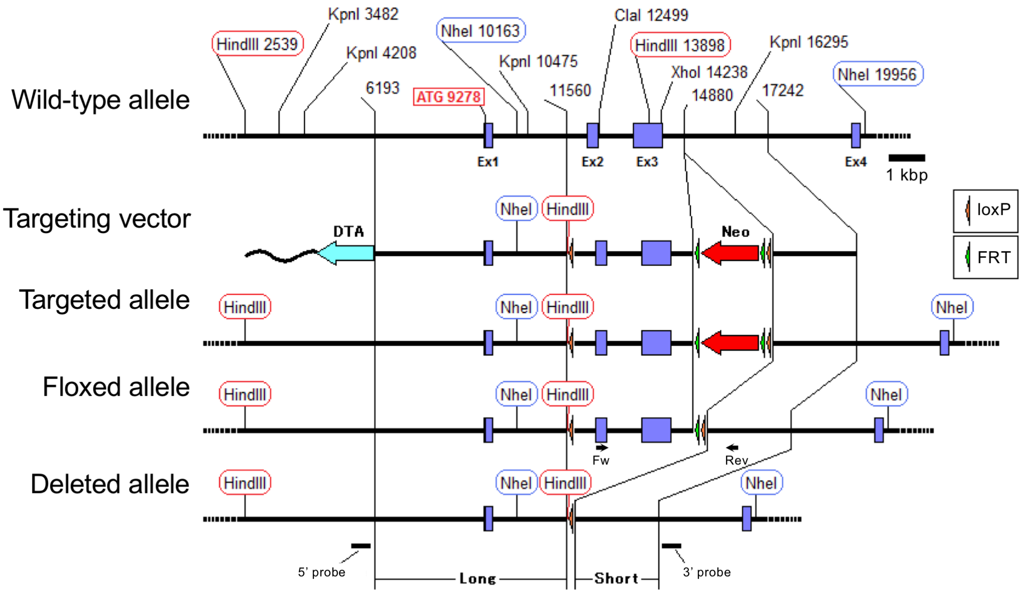

Figure S1. Generation of adipocyte-specific GPRC6A knockout (adG6AKO) mice. Schematic representation of the wild-type Gprc6a allele, the targeting vector, and the targeted (Gprc6aneo-fl), floxed, and deleted alleles. Exons (Ex) are shown as numbered boxes. The targeted allele contains a selection cassette comprised of a neomycin resistance gene (Neo, red arrow) flanked by flippase recognition target (FRT) sites (green triangles), as well as two loxP sites (pink triangles) flanking exons 2 and 3 and the selection cassette. The 5' and 3' external probes for Southern blot analysis are indicated by thick black lines, and HindIII and NheI restriction sites are shown. DTA, diphtheria toxin A gene.

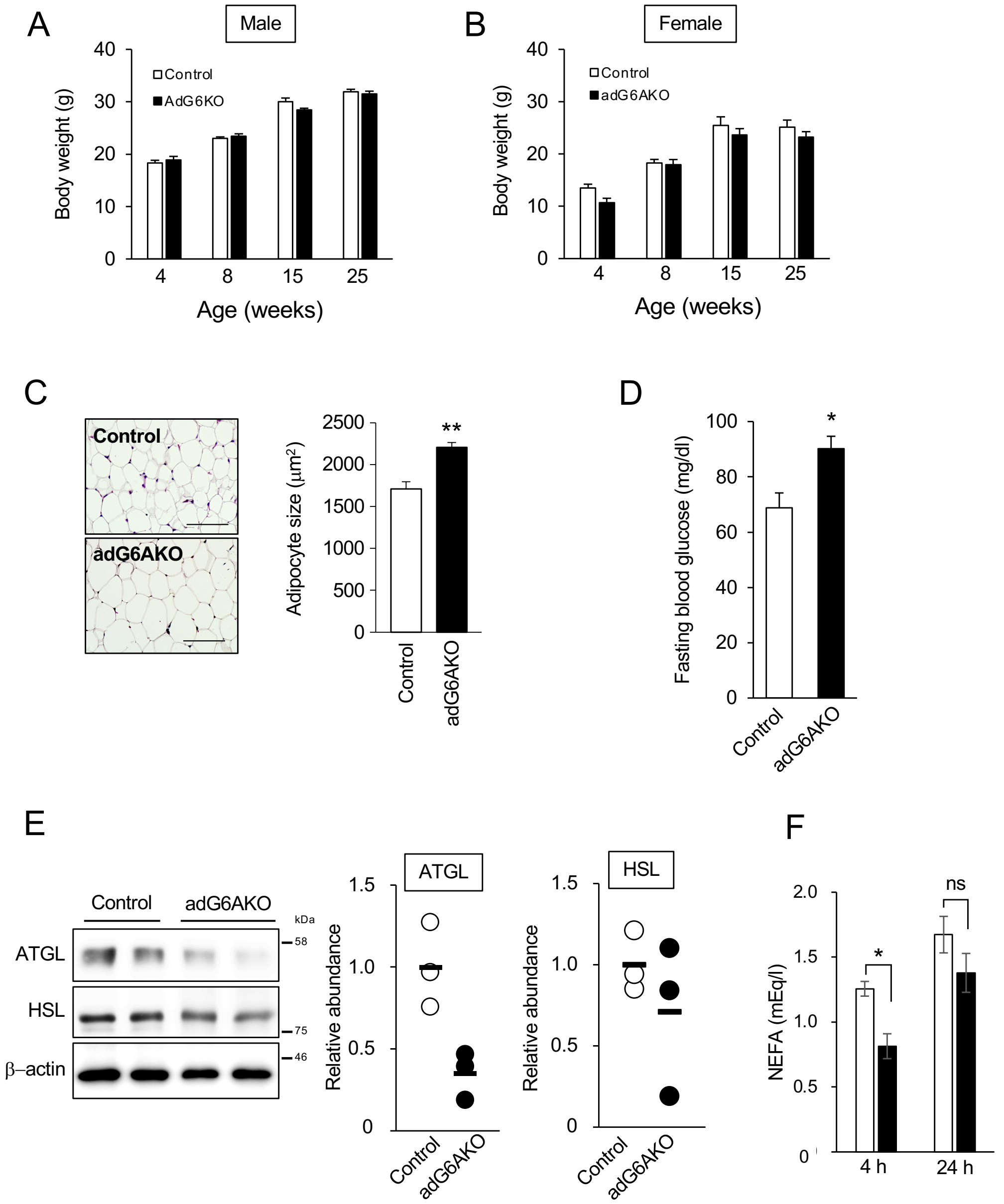

Figure S2. Characterization of adG6AKO mice fed a normal diet. *A* and *B*, Body weight of control and adG6AKO male (*A*) and female (*B*) mice at the indicated ages. *C*, HE staining of eWAT from control and adG6AKO mice at 25 weeks of age (left), and adipocyte area determined for 50 adipocytes per slide and at least three sections for each mouse (right). Bars, 100 μm. *D*, Blood glucose levels after food deprivation for 16 h in control and adG6AKO mice at 25 weeks of age. *E*, Immunoblot analysis of ATGL and HSL in eWAT of control and adG6AKO mice at 25 weeks of age. Representative blots for two mice of each genotype are shown, as well as quantitative data for relative ATGL or HSL protein abundance normalized to that of β-actin from multiple blots. *F*, Serum NEFA levels after food deprivation for 4 or 24 h in control and adG6AKO mice at 25 weeks of age. All quantitative data are the mean + SEM for 8–10 mice. $^*P < 0.05$, $^{**}P < 0.01$ versus the corresponding value for control mice or for the indicated comparisons (Student's *t*-test).

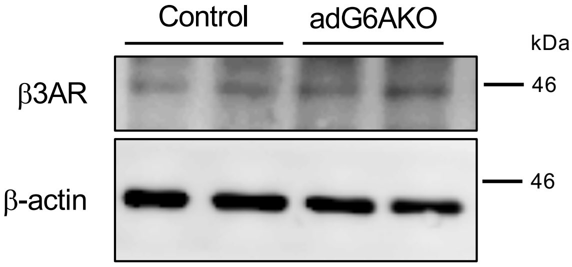

Figure S3. Comparison of β3-adrenergic receptor in WAT from control and adG6AKO. Membrane fractions of epididymal WAT from control and adG6AKO were analyzed for immunoblot analysis using β3-adreneric receptor-specific antibody. Whole lysates before fractionation were used to detect β-actin as an internal control. β3AR; β3-adreneric receptor.

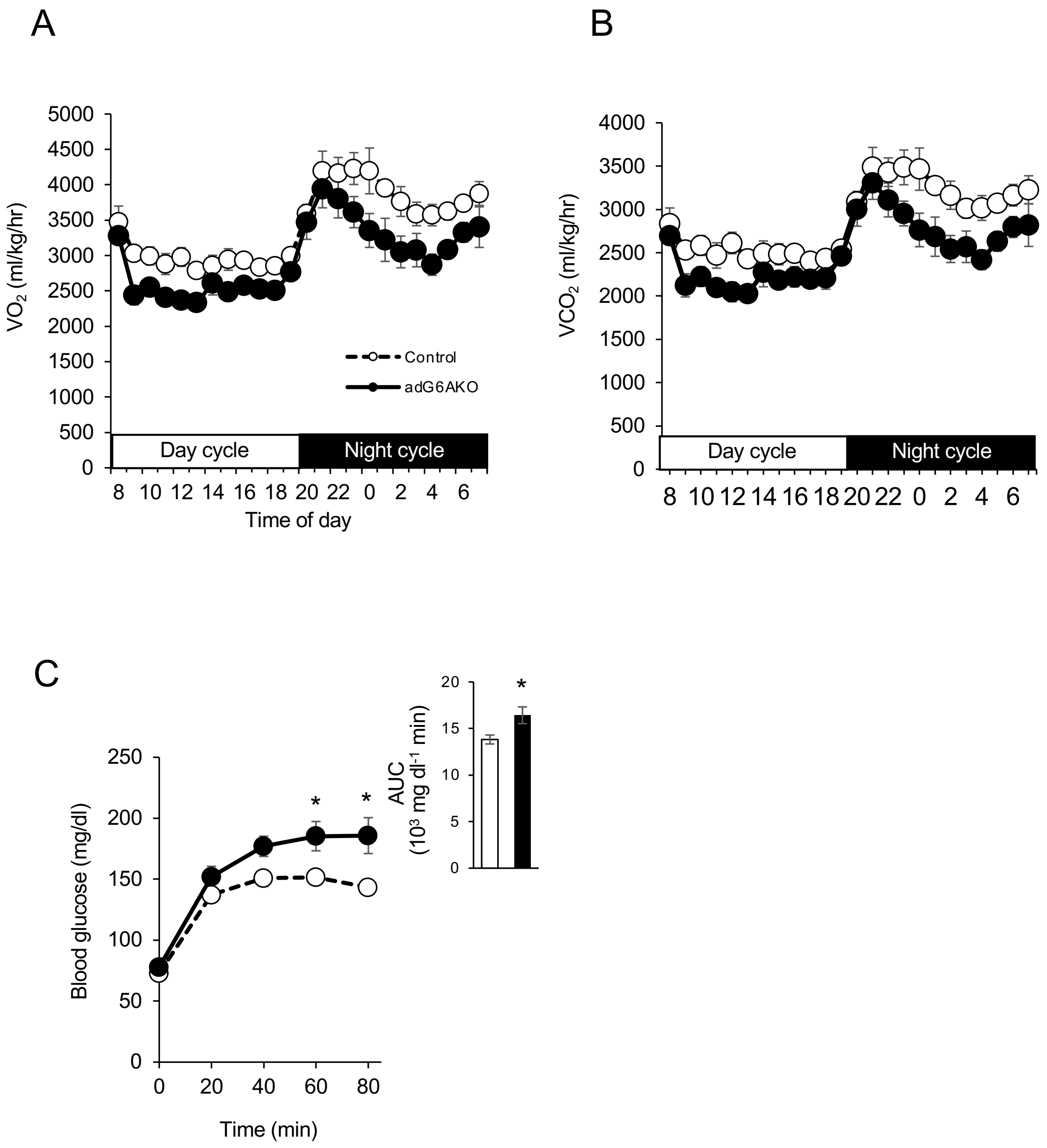

Figure S4. Metabolic analyses. *A* and *B*, Oxygen consumption (VO_2) (*A*) and carbon dioxide production (VCO_2) (*B*) were assessed by an indirect calorimetric system over a 24-h period with a 12-h light/dark cycle. *C*, Pyruvate tolerance test (PTT) for control and adG6AKO mice fed an HFS for 17 weeks. The area under the curve (AUC) is shown in each inset. All data are the mean ± SEM for 10 mice per group. *P < 0.05 versus the corresponding value for control mice (two-way ANOVA followed by Bonferroni's post hoc test.

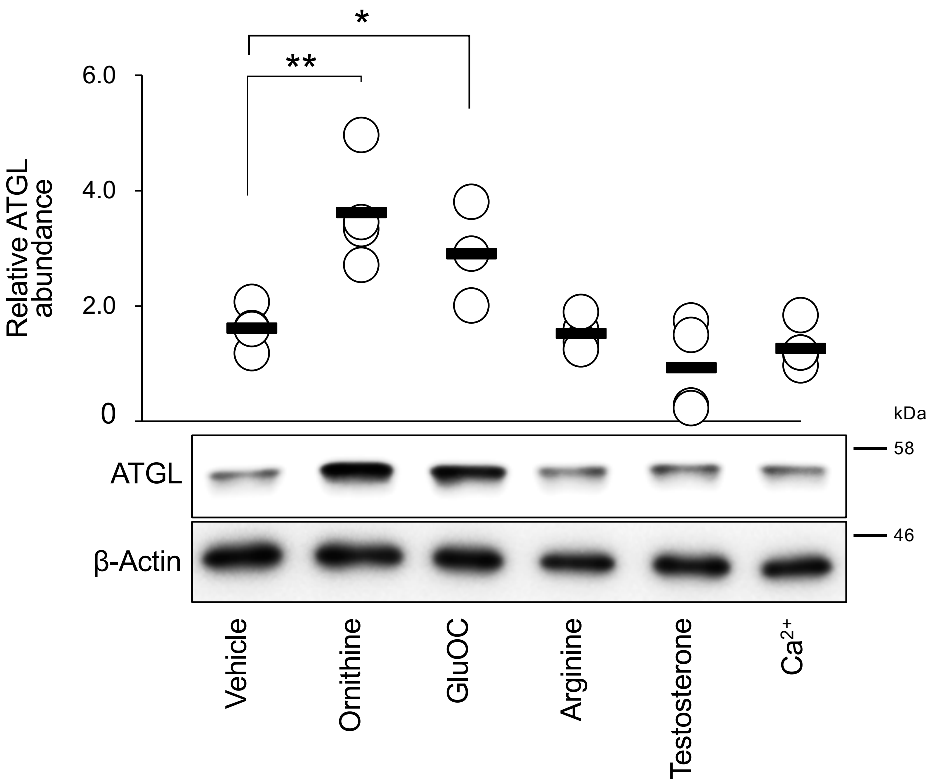

Figure S5. Effects of GPRC6A ligands on ATGL expression in 3T3-L1 adipocytes. 3T3-L1 adipocytes stimulated with ornithine (1 mM), GluOC (2.5 ng/mL), arginine (1 mM), testosterone (10 nM), or Ca^{2+} (1 mM) for 8 h were subjected to immunoblot analysis with antibodies to ATGL. Representative blots as well as quantitative data (mean + SEM) are shown for relative ATGL abundance normalized to that of β-actin from three independent experiments. *$P < 0.05$, **$P < 0.01$ (one-way ANOVA).

Table S1. Primer sequences for analysis of gene expression. Sequences of PCR primers (F, forward; R, reverse) are shown for the indicated genes. GenBank accession numbers and PrimerBank IDs (http://pga.mgh.harvard.edu/primerbank/index.html) are indicated.

Gene		Sequence (5'–3')	GenBank Accession	PrimerBank ID
Gprc6a	F	GCTTTACTCTCTGTGTCTCC	NM_153071	
	R	GGGTCAAAACTGAAAGCTAGC		
Pnpla2	F	GGGTGCGCTATGTGGATGG	NM_001163689	254826779c3
	R	CTCTCGCCTGAGAATGGGG		
Lipe	F	GGCTTACTGGGCACAGATACCT	NM_010719	
	R	CTGAAGGCTCTGAGTTGCTCAA		
Plin2	F	CAAGCACCTCTGACAAGGTTC	NM_001113471	164698412c2
	R	GTTGGCGGCATATTCTGCTG		
Pparg	F	CCAGAGTCTGCTGATCTGCG	NM_001127330	
	R	GCCACCTCTTTGCTCTGCTC		
Klf15	F	GAGACCTTCTCGTCACCGAAA	NM_023184	
	R	GCTGGAGACATCGCTGTCAT		
Cd36	F	GGAGCCATCTTTGAGCCTTCA	NM_001159556	227116348c3
	R	GAACCAAACTGAGGAATGGATCT		
Slc27a2	F	GATGCCGTGTCCGTCTTTTAC	NM_011978	113374153c2
	R	GACTTCAGACCTCCACGACTC		
Fas	F	GGAGGTGGTGATAGCCGGTAT	NM_007988	30911099a1
	R	TGGGTAATCCATAGAGCCCAG		
Srebp1c	F	GTCAAAACCAGCCTCCCAAG	NM_011480	
	R	CAGTCCCCGTCCACAAAGA		
Tnf	F	CAGGCGGTGCCTATGTCTC	NM_013693	133892368c1
	R	CGATCACCCCGAAGTTCAGTAG		
Il6	F	GCTACCAAACTGGATATAATCAGGA	NM_031168	
	R	CCAGGTAGCTATGGTACTCCAGAA		
Adgre1	F	GGAGGACTTCTCCAAGCCTATT	NM_001355722	
	R	AGGCCTCTCAGACTTCTGCT		
Actb	F	GGCTGTATTCCCCTCCATCG	NM_007393	6671509a1
	R	CCAGTTGGTAACAATGCCATGT		

Table S2. Antibodies used for immunoblot analysis.

Antibody (Cat#)	Dilution	Supplier
ACOX1 (ab184032)	1/1000	Abcam, Cambridge, MA, USA
ATGL (#2138)	1/1000	Cell Signaling Technology, Danvers, MA, USA
Adrenaline receptor β3 (GTX54925)	1/1000	GeneTex, Irvine, CA, USA
FoxO1 (#2880)	1/1000	Cell Signaling Technology
GPRC6A (SAB4500879)	1/1000	Santa Cruz Biotechnology, Santa Cruz, CA, USA
HSL (#18381)	1/1000	Cell Signaling Technology
Hsp40	1/1000	StressGene Biotech, British Columbia, Canada
IRF4 (#4948)	1/1000	Cell Signaling Technology
MCAD (ab110296)	1/1000	Abcam
PPARα (ab24509)	1/1000	Abcam
Perilipin (#9349)	1/1000	Cell Signaling Technology
Phospho-HSL (Ser^{563}) (#4139)	1/1000	Cell Signaling Technology
Phospho-perilipin (4856)	1/1000	Vala Sciences, San Diego, CA, USA
UCP1(#14670)	1/1000	Cell Signaling Technology
β-actin (A5316)	1/5000	Sigma, St. Louis, MO, USA
